# Predicting tissue specific transcription factor binding sites

**DOI:** 10.1186/1471-2164-14-796

**Published:** 2013-11-15

**Authors:** Shan Zhong, Xin He, Ziv Bar-Joseph

**Affiliations:** 1 Lane Center for Computational Biology, School of Computer Science, Carnegie Mellon University, Pittsburgh, PA, 15213, USA

## Abstract

**Background:**

Studies of gene regulation often utilize genome-wide predictions of transcription factor (TF) binding sites. Most existing prediction methods are based on sequence information alone, ignoring biological contexts such as developmental stages and tissue types. Experimental methods to study *in vivo* binding, including ChIP-chip and ChIP-seq, can only study one transcription factor in a single cell type and under a specific condition in each experiment, and therefore cannot scale to determine the full set of regulatory interactions in mammalian transcriptional regulatory networks.

**Results:**

We developed a new computational approach, PIPES, for predicting tissue-specific TF binding. PIPES integrates *in vitro* protein binding microarrays (PBMs), sequence conservation and tissue-specific epigenetic (DNase I hypersensitivity) information. We demonstrate that PIPES improves over existing methods on distinguishing between *in vivo* bound and unbound sequences using ChIP-seq data for 11 mouse TFs. In addition, our predictions are in good agreement with current knowledge of tissue-specific TF regulation.

**Conclusions:**

We provide a systematic map of computationally predicted tissue-specific binding targets for 284 mouse TFs across 55 tissue/cell types. Such comprehensive resource is useful for researchers studying gene regulation.

## Background

To reconstruct and model transcriptional regulatory networks (TRNs) we need to know the genome-wide binding sites of transcription factors (TFs) [1,2]. Chromatin immunoprecipitation(ChIP) followed by microarray (ChIP-chip) [3] or sequencing (ChIP-seq) [4] has been extensively used to study the *in vivo* binding locations of individual transcription factors and cofactors in a wide range of species and tissues [1,2,5-9]. Despite their popularity, such methods can only study a single TF in a single cell type, under a specific condition, in each experiment. Thus, it is difficult to use these methods to obtain a comprehensive understanding of the complicated mammalian TRNs. These networks can involve hundreds or thousands of TFs whose activities change across different tissues and conditions. Using computational methods to integrate other genomic resources in order to predict tissue-specific transcription factor binding is therefore an important research challenge.

Several methods have been developed to use *in vitro* data characterizing TF binding specificities to identify TF binding sites across the genome. Specifically, data from universal protein binding microarray (PBM) [10,11] is often used for such analysis. PBM is capable of analyzing the interaction of a sequence-specific TF with tens of thousands of short DNA sequences (probes) in a single experiment, and thus provides a highly detailed picture of TF-DNA interactions. It has been successfully applied to reveal the binding profiles of hundreds of TFs in yeast [12], worm [13], mouse [14] and arabidopsis [15]. Some of the proposed methods for using PBM data represent TF binding preference by position weight matrices (PWMs) [11,16,17]. However, PWMs, although popular due to their simplicity, assume independence between positions, an assumption which may not hold in many cases [14,18,19]. In contrast, more sophisticated models (e.g. using *k*-mers) may better represent the full binding profiles of TFs, without loss of information from using PWM. For instance, it has been suggested that many TFs have more than one binding preference [14] and these are easier to represent using k-mers.

While *in vitro* data provides important information regarding binding specificities, such data is context independent. Actual binding is highly dependent on tissue-specific conditions including chromatin accessibility, the presence of co-factors, etc [20]. Recently, a number of studies have reported that epigenetic information including certain histone modifications and hypersensitivity to DNase I cleavage correlate with TF binding *in vivo*[21,22]. Moreover, functional TFBSs tend to be under stronger negative selection, leaving a "phylogenetic footprint" in the genomic sequences. Several methods for predicting *in vivo* TF binding sites have attempted to combine such information with PWMs to predict global binding preferences [23-26]. However, as mentioned above, PWM may not be the best representation of TF binding. As we show, by using a model that retains the dependence between positions in the motif we can improve upon methods that integrate epigenetic and PWM data. In addition, none of these methods have so far been applied to elucidate the complete set of targets for TFs across a large number of tissues.

To predict accurate tissue-specific TFBS, we integrate multiple types of genomic data. The first part of our model is a biophysically-motivated *k*-mer based method for analyzing PBM data, which allows secondary binding profiles and nucleotide dependencies in different positions of the TF binding sites. Next, we develop a new method, PIPES (*p*robabilistic *i*ntegration of *P*BM, *e*pigenetics and *s*equence data), to combine the results from the PBM model with DNase I hypersensitivity (DHS) data and evolutionary conservation data to predict tissue-specific TFBS *in vivo*. We demonstrate that such an integrative model significantly boosts context specific prediction results compared with using PBM data alone. We also show that PIPES improves upon other methods developed for integrating data to predict TFBS [24,26], in some cases significantly so. Finally, we created a resource for tissue-specific TRNs using PBM data for 284 mouse TFs from UniPROBE [27] and DNase I hypersensitivity data for 55 mouse tissue/cell types from the mouse ENCODE project [28]. We predict the activities of TFs across different tissues, and, as we show, many of these predictions agree with current knowledge regarding tissue-specific roles of TFs. Our tissue specific activity predictions are also supported by global analysis of TF expression data. The comprehensive resource of TF binding sites we built thus provides a reference map for understanding complex gene expression patterns.

## Results

**Figure 1:**
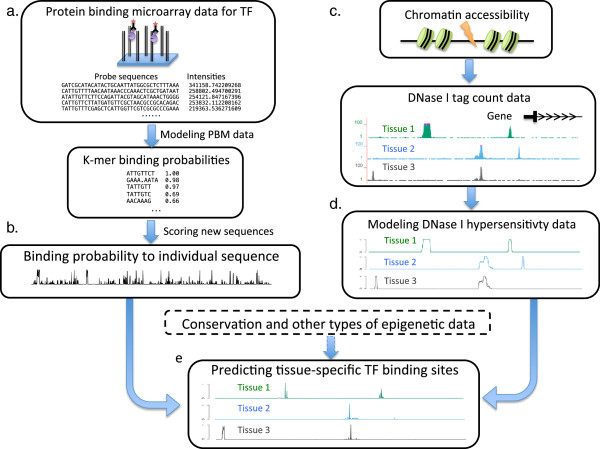
**Overview of PIPES.** **(a, b)** Starting with raw PBM data for a TF represented as fluorescence intensities to each individual probes, we first infer binding probabilities to individual short *k*-mers, and then a given sequence can be scored by such inferred binding probabilities. **(c, d)** To predict *in vivo* TF binding, we take as input the tissue-specific DNase I hypersensitivity data (tag counts) and convert them to probabilities that represent chromatin accessibility for each position in the genome at each tissue/cell types. **(e)** The PBM data, DNase data and other types of data including sequence conservation are combined using an integrative model. See Methods for details.

An overview of our PIPES method is shown in Figure 1. Our model has two components: the left part of the figure shows our model for the PBM data, and the right part our model of epigenetics and conservation data. Starting with raw fluorescent intensities measured by PBM, we first infer binding probabilities to each individual *k*-mer with a biophysically-motivated model (Figure 1a). This information, based on PBM alone, can be used to score a sequence for potential TFBS (Figure 1b). Next, we use tissue specific DNase I hypersensitivity data to determine chromatin accessibility (Figure 1c, d), and combine such information with sequence conservation and the PBM derived scores to predict *in vivo* binding sites (Figure 1e).

### *K*-mer based PBM analysis can accurately infer TF binding specificities

While a number of methods have been suggested to use PBM data for predicting TF binding sites (in most cases using PWMs), we decided to extend *k*-mer based methods using a biophysically-motivated model (Figure 1a). *K*-mer based methods were shown to achieve the best performance among several techniques for the analysis of PBM data [29]. Such methods allow an intuitive representation of potential alternative binding motifs and can account for dependency among positions in a motif. We use lasso regression with positive constraints to learn model parameters that represent binding probabilities to individual *k*-mers, where *k* is determined as part of the learning procedure. This results in a sparse model with relatively few *k*-mers having nonzero binding probabilities. On average, the number of *k*-mers with non-zero probabilities is 398.4 ± 253.2 across 284 mouse TFs with PBM data available. The model combines the benefits of recent PWM-based biophysical methods (for example, BEEML-PBM [16]) with the ability of PBMs to capture dependencies between positions in a given motif (see Methods and Supplementary Methods in Additional file 1 for details).

**Figure 2:**
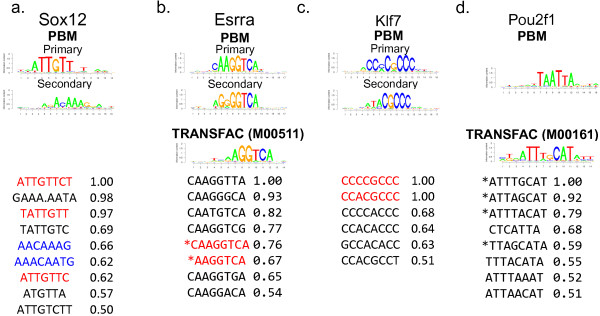
**Top inferred** ***k*-mer binding probabilities for (a) Sox12, (b) Esrra, (c) Klf7 and (d) Pou2f1.** The S&W PWMs and TRANSFAC motifs (when available) for these four factors are also shown. *k*-mers are colored according to whether they match the consensus sequences of the primary S&W PWM (red) or secondary S&W PWM (blue). *k*-mers matching the TRANSFAC motifs are indicated by a "*" in the front. Only k-mers with coefficients above 0.5 (normalized so that the maximum is equal to 1) are shown.

We illustrate the results of our PBM model using four TFs including Sox12, Esrra, Klf7 and Pou2f1. Figure 2 presents the PWMs derived from the PBM data for these TFs by the Seed-and-Wobble algorithm [11] (denoted as S&W PWMs), PWMs in TRANSFAC [30] for the corresponding TFs when available, and all the *k*-mers estimated by our model that have binding probabilities above 0.5. For S&W PWMs, when a secondary binding preference was derived [14], both the primary and secondary PWMs are shown. As can be seen, our *k*-mer model does well for this data. For Sox12, the learned *k*-mers match well with both the primary and secondary S&W PWMs (Figure 2a). For Esrra and Klf7, *k*-mers matching the consensus sequences of the primary S&W PWMs, respectively, are predicted to have high binding probabilities (Figure 2b and c), whereas *k*-mers matching the secondary S&W PWMs have lower predicted binding probabilities ranging from 0.2 to 0.44 (not shown in the figure). In the case of Pou2f1, none of the inferred top *k*-mers match the S&W PWM (Figure 2d). However, many of these top *k*-mers closely match the consensus sequence of the TRANSFAC motif for Pou2f1 derived from literature evidence (Figure 2d). Overall, these results support the use of a biophysically-motivated model: the binding probabilities of *k*-mers are largely consistent with the results from independent methods and known motifs from TRANSFAC.

To test our PBM model, and as a baseline, we next used the inferred binding probabilities to predict *in vivo* TF binding. We collected 11 published mouse ChIP-seq datasets for which the PBM data for the same TF or for a TF with a similar DNA-binding domain is available (Additional file 2). From each ChIP-seq dataset, the top 3000 peaks with highest enrichment are extracted, and the 600bp genomic regions centered on the reported peaks are used as the positive sequences bound by the TF. Then, 600bp sequences that (1) are upstream of and (2) 300bp apart from each positive sequence, and (3) do not overlap with any other positive sequences, are used as negative sequences. We also explored two alternative options for constructing negative sequences, including using size-matched random promoter sequences and randomly generated sequences. The choice of negative sequence sets makes very little difference on the AUC values, so we will only report the results based on the first negative set here (see Supplementary Results in Additional file 1, and Additional file 3 for details). We compared our PBM model with seven other methods that predict affinities of TF binding to given sequences, and the different methods are evaluated using areas under the ROC curve (AUC) as a measure of their abilities to correctly classify the two sets of sequences (see Methods, Supplementary Results in Additional file 1 and Figure S1 therein for details).

The results indicate that the performance of our PBM method is at least comparable, and in some cases better than, previous methods. For 4 of the 11 TFs we tested (Esrrb, Sox2, Oct4 and Crx), our method improved over all other methods. In all other cases, the AUC of our method ranks within the top 4 (Supplementary Results in Additional file 1 and Additional file 1: Figure S1 therein). Notably, in such cases, none of the other methods consistently achieves the best AUC. The PWM-based and E-score based methods tend to work well for some cases (for example, BEEML PWM for Klf7 and Max E-score for Srf), but for others their performance is not as good. Overall, our PBM model has the highest average AUC over the 11 TFs tested (Supplementary Results in Additional file 1, and Additional file 4).

To further assess the benefits of using PBM to derive TF binding specificities, we use two other collections of PWMs for AUC evaluation. The HOMER PWMs [31] were derived from ChIP-seq datasets, while the JASPAR PWMs were from multiple sources (including ChIP-seq, literature curation and PBM data). Overall, the results from the JASPAR PWMs are very similar to those obtained from the Seed and Wobble PWMs, and both are weaker than our method. The use of HOMER PWMs lead to better overall performance. However, given that HOMER trains the PWMs from ChIP-seq data and the same datasets may be used for evaluation, this is clearly not a fair comparison. We did notice that for some TFs, our method outperforms HOMER PWMs (e.g. Max: 0.809 vs 0.757). The full details are shown in Supplementary Results in Additional file 1 and Additional file 4.

### Integrated model of PBM and DNase I hypersensitivity data significantly improves TFBS prediction accuracy

PBM data, although powerful, only measures *in vitro* binding. Therefore, even when using sophisticated methods, the ability to predict *in vivo* binding based on PBM data is limited. DNase I hypersensitive (HS) sites are regions of chromatin that are very sensitive to DNase I cleavage [32], and previous studies have shown that such hypersensitivity correlates with TF binding [21,22]. To better predict tissue-specific *in vivo* binding sites, we developed PIPES, a probabilistic graphical model for integrating DNase I HS data with PBM data. For windows containing a 36bp genomic region ("*site*"), we assume the chromatin of the site could exist in two states: *open* or *closed*, and that only in the open state the chromatin is accessible to binding by a TF. We infer the chromatin state by using a mixture model for the DNase HS data: the open state should be associated with higher tag densities from the DNase data, and the closed state with lower densities. The *in vivo* occupancy of a site is then estimated as the probability of binding *in vitro* estimated using result from the PBM model multiplied by the probability that the site is in an open state inferred from the DNase HS data (see Methods for a detailed description of PIPES).

**Figure 3:**
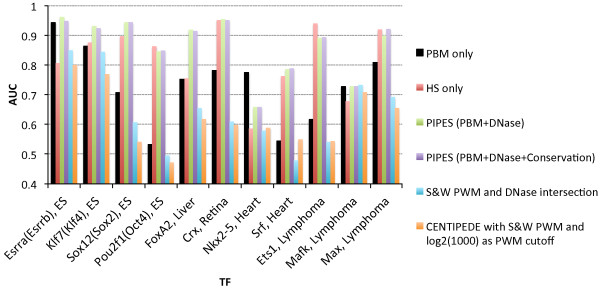
**AUCs of different integrative methods for predicting** ***in vivo* TF binding.** The first four bars are from our *k*-mer PBM data analysis method (black), simple baseline using only DNase data (red), or from PIPES (green and purple). Cyan bars: Simple overlapping approach that overlays PWM matches with DNase data. Orange bars: CENTIPEDE using S&W PWMs to scan sequences and an odd log score of log2(1000) as cutoff. See main text and also Additional file 1 for more details.

Figure 3 presents the AUCs from applying PIPES to predict *in vivo* TF binding in the corresponding tissues for the same 11 TFs studied in the previous section. Compared with using PBM data alone (black bars), the incorporation of DNase I HS data in the corresponding tissues (green bars) improves performance for 10 of the 11 TFs. Overall, the improvement of AUC from adding DNase HS data across all TFs is statistically significant (*p* = 0.0049, one sided Wilcoxon rank-sum test). The biggest improvements are seen for TFs for which the results when using only PBM data are relatively poor. For example, when predicting Srf binding sites in heart, even the best methods analyzed above achieve an AUC only slightly better than random (Figure 3). By integrating PBM and DNase data, the performance of our method is improved by 46% from 0.539 to 0.787 (Figure 3). Similar improvement is also observed for Oct4 (from 0.532 to 0.847). As another baseline, we evaluated the AUCs from using DNase HS data alone. Somewhat surprisingly, this feature alone seems quite discriminative (Figure 3, red bars; see also Additional file 4): the mean AUC is 0.822. Nevertheless, in 8 out of 11 cases, our model using both DNase and PBM data improves these baseline results and its mean AUC is also higher at 0.866. We also point out that in practice it is not appropriate to use the HS data alone to predict binding sites for a TF as the predictions would not be specific to the TF of interest.

In addition to DNase I hypersensitivity data, *bona fide* TF binding sites are usually under evolutionary pressure and therefore more conserved [33,34]. We thus further extended PIPES to incorporate phastCons scores [35] for each site (Methods). Performance of the full model that incorporates PhastCons information is shown in Figure 3 (purple bars). As can be seen, while in some cases adding the conservation information very slightly improves performance (for example for Srf and Oct4), overall using conservation data does not lead to a significant improvement in prediction accuracy. When the DNase HS data is not available, using PhastCons in addition to the PBM data provides a slight improvement of the AUCs from using PBM alone (improving the results for 8 out of 11 TFs, Additional file 4). The average AUC is increased by 1%, and for some TFs, the improvement can be quite significant (e.g. Srf, AUC changes from 0.539 to 0.592 by adding PhastCons).

Finally, we compare PIPES with recent methods proposed for integrating DNase and motif information to predict TFBS. The first method we compare against, termed 'Intersection", was used by Neph et al. [26]. This method intersects sites that have high-scoring PWM matches for a TF with DNase HS sites to predict in vivo binding (Supplementary Methods in Additional file 1). We also compare PIPES with CENTIPEDE [24]. CENTIPEDE uses a probabilistic model to integrate the prior information of putative sites, such as sequence conservation and matches to PWMs, with the epigenetic data to predict binding sites. While probabilistic, CENTIPEDE does, however, rely on a stringent cutoff for PWM match scores to achieve low false positive rates.

The results are presented in Figure 3. As can be seen, the intersection method leads to AUCs that are significantly lower than the ones obtained by our method for all 11 TFs (cyan bars, *p* = 9.77 · 10^-4^, one sided Wilcoxon rank-sum test), indicating that strict cutoffs (as opposed to probabilistic integration) may lead to a high rate of false negatives. Similarly, using the default settings for CENTIPEDE led to AUC scores that are much lower than ours (Figure 3, orange bars). To further explore this, we varied the setting of CENTIPEDE, including using different PWMs and a range of cutoffs for defining putative binding sites, but the results remained the same (See Supplementary Methods and Results in Additional file 1, and Additional file 4). The difference in AUCs is highly significant: *p* = 4.88 · 10^-4^ (one sided Wilcoxon rank-sum test) using the best combination of PWM and cutoff for CENTIPEDE. These results indicate that our PIPES model, which relies on *k*-mer based representation and avoids strict cutoffs, can improve *in vivo* predictions of TFBS.

We performed additional analysis to the integration model. For these, we replace the binding probabilities predicted from the *k*-mer model, with those predicted when using PWMs. Three different versions of PWMs were used: the Seed-and-Wobble PWMs and RAP PWMs learned from the same PBM data, and the JASPAR PWMs. In all cases, the AUCs are substantially higher than the ones from the Intersection method and CENTIPEDE (below or close to the full PIPES model). These results indicate that the probabilistic integration step alone is enough to improve upon prior methods (Additional file 4). The usefulness of the *k*-mer based analysis provides additional advantage, as independently demonstrated in the earlier section.

### Combining PBM and DNase data enables the prediction of tissue-specific TF activities

The recently released mouse ENCODE project data provides DNase I hypersensitivity data for more than 50 mouse tissue/cell types (Methods). We set out to combine the PBM data for 284 mouse TFs in UniPROBE with such DNase data to predict tissue-specific TF targets and determine tissue-specific TF activities (Methods).

**Figure 4:**
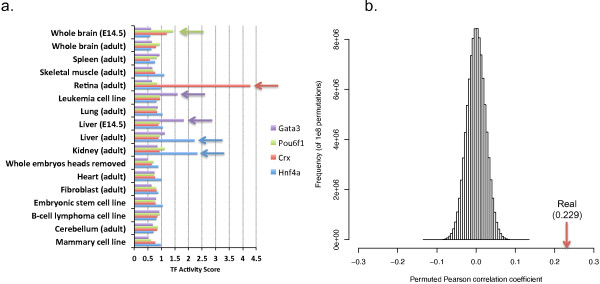
**Results for tissue-specificity TF activity prediction.** **(a)** Predicted tissue activity scores for 4 TFs across 18 representative tissue/cell types. Arrows indicate known functions of the TF in the corresponding tissue as supported by literature evidence. In all cases, the highest activity score matches a known tissue for the factor. **(b)** Pearson correlation coefficients between tissue specific expression experiments and the activity level predicted by our method. The distribution is based on 10^8^ permutations of the activity scores. The value from the real predictions is indicated by the arrow on the right.

Identifying TFs that are highly active in specific tissues is useful for determining the function of such TFs, and serves as an initial step for reconstructing the tissue-specific transcriptional regulatory networks. We predict how likely a TF is functional in any given tissue/cell type with an activity score for each TF-tissue pair. Our hypothesis is that if the TF is active in a tissue, it will bind a number of target sequences, thus the putative TF binding sites will be overrepresented in the DNase HS regions (see Methods for details). A higher activity score indicates that the TF is more active in the corresponding tissue (the expected value is 1 for non-active TFs). We use a binomial test to assess the statistical significance of the activity scores (see Methods). The complete results, including activity scores and *p*-values, are provided in Additional file 5. In Figure 4a we illustrate these results by focusing on the activity scores calculated for 4 TFs (Gata3, Pou6f1, Crx and Hnf4a) across 18 representative tissue/cell types. Gata3 is known to function in mouse fetal liver haematopoiesis [36], and its expression had also been observed in leukemia cells [37]. Our results are in good agreement with the prior knowledge regarding Gata3’s activity: the top two tissues predicted for Gata3 are E14.5 liver cells and the adult leukemia cell line. Similarly the top tissue for Pou6f1 is E14.5 whole brain, in agreement with its known role in brain development [38]. Crx is an important TF for regulating photoreceptor genes in retina [39,40], and our method correctly determined that its activity score in that tissue is the highest. Finally, Hnf4a is a well known master regulator of liver- and kidney-specific genes [41,42], as correctly predicted by our method. While we only show 18 tissues, for all four TFs the correct tissues shown in Figure 4a have the highest scores among all 55 tissues we tested (Additional file 5).

To more globally validate these tissue-specific TF activities, we compared the correlation between our predicted TF activity scores and mRNA levels for the same TFs in the corresponding tissue (measured by qRT-PCR [43]). Eight tissues and 222 TFs that are common to both datasets are used (Additional file 6). Even though the two types of data (PBM and DNase vs. expression) measure completely different aspects of cellular activity, we observe a Pearson correlation coefficient of 0.229, which is highly statistically significant (*p* < 10^-8^, permutation test, Figure 4b). Since many TFs are only post-transcriptionally regulated, such a significant correlation provides strong support to the predictions computed by our method.

### Existing literature strongly supports predicted TF activities in several tissues

To further validate our predictions and investigate their potentials to lead to new biological insights, we took a closer look at the TFs predicted to be active in the adult liver tissue. The top five such predictions are shown in Table 1A (*p* < 10^-100^ for all, binomial test). Besides Hnf4a discussed above, Rara, Nr2f2, Rxra and Tcf7 are all known to either regulate liver-specific genes or are involved in maintaining liver metabolism and homeostasis (Table 1A). The 7*th* ranked factor Tcf7l2 (activity score of 1.38) was linked to type 2 diabetes risk in previous studies using SNP data [44], but the mechanism for its involvement was unclear. Our result indicates that it may have a regulatory role in liver metabolism. Indeed, a very recent study confirms its role in regulating key liver-specific metabolic genes [45]. Our result also assign a high liver activity score to Cutl1 (1.36, rank 8/284). Cutl1 was a known transcriptional repressor of terminal differentiation genes in several cell lineages including hepatocyte [46]. Recently, Cutl1 was identified as target of the liver-specific microRNA miR122 and a central mediator of the effects caused by the deregulation of miR122 in hepatocellular carcinoma [47]. Further down the list, Foxa2 (1.32, rank 10/284) is known to regulate lipid metabolism and ketogenesis related genes in liver [48], and Lef1 (1.31, rank 11/284) is a prognostic biomarker for liver metastasis in colorectal cancer. Other TFs ranked within the top 20 for liver include Tcf1 and Tcf2, members of the T-cell factor (Tcf) family that are critical for hepatocyte metabolism and function [49,50]; Bhlhb2, which is involved in the regulation of lipogenesis in liver [51]; and Hmbox1, whose expression levels was shown to be reduced in liver cancer compared with surrounding normal tissues [52]. Overall, our predicted set of liver regulators is comprehensive, spanning several different classes of liver related activities including glucose and lipid metabolism and cancer, and including both repressors and activators. In addition, Table 1B and C presents the top 5 predicted TFs for two more tissues (retina and B cell, *p* < 10^-100^ for all, binomial test). As can be seen, for almost all of these TFs there is strong support for their tissue-specific activity in the predicted tissue.

**Table 1:** Top five predicted TFs for liver, retina and B cell

**TF**	**Score**	**Known functions in the corresponding tissue**
**A. Liver**		
Hnf4a	2.22	Essential for maintaining hepatic gene expression and lipid homeostasis [41]
Rara	1.90	Important in maintaining liver homeostasis, and its disruption is linked to hepatocarcinogenesis [57]
Nr2f2	1.56	Expressed in liver, and known to regulate liver-specific genes [58]
Rxra	1.45	Important role in liver metabolism [59]
Tcf7	1.44	Downstream regulator in Wnt signaling which is critical in liver physiology and pathology [60]
**B. Retina**		
Crx	4.28	Regulates photoreceptor gene expression [40]
Pitx3	4.26	Required for normal retina formation in Xenopus and zebrafish [61,62]
E2F3	4.06	Involved in retina progenitor cell development [63]
Pitx2	3.92	Pitx2-deficient mouse exhibits ocular abnormalities [64]
Gsc	3.89	Unknown function in retina.
**C. CD19+ B cell**		
Sfpi1	2.09	Essential regulator of B-cell differentiation [65]
Pou2f2	2.08	Required for T-cell independent B cell activation [66]
Spic	1.98	Promotes B cell differentiation [67]
Pou2f3	1.94	Unknown function in B cell, but has almost the same binding preference as Pou2f2
Elf4	1.70	Regulates proliferation of B cells [68]

## Discussion

A number of recent projects including ENCODE [53], modENCODE [54,55] and the Roadmap Epigenomics Project [56] have generated large amounts of genomic data. An important research goal is to translate these resources into accurate, tissue and condition-sensitive, molecular-level networks. Constructing tissue-specific maps of TF binding sites is a central part of this overall research effort. Using several different datasets and a novel computational strategy, PIPES, we demonstrated that such high-quality computational predictions can be obtained. We used PIPES to compile a resource that includes comprehensive predictions for more than 200 TFs across 50 tissues.

A recent benchmark study that compared many methods for analyzing PBM data concluded that PWM-based methods work as well as other models for predicting TFBS [69]. Our results differ from these previous studies. This could either be the result of the Lasso based method we have used or the specific dataset we used for the comparison. Additional work is required to reach a definitive conclusion regarding the importance of independence assumption used by PWMs when modeling TFBS. Here we focused on integrating a number of datasets for predicting TFBSs. For our biophysical approach, using a *k*-mer based method allowed us to capture both the dependency among positions within the binding site as well as multiple different motifs for a single TF. Such method worked well in classifying bound and unbound sequences from in vivo ChIP-seq data for many TFs. Moreover, integrating PBM data with chromatin accessibility from DNase I HS data greatly improves the accuracy of TF binding predictions. Several recent papers explored related ideas. Chromia [70] used a hidden Markov model to combine sequence-specific TF binding with histone modification data, but their predictions were based on PWM scoring and only focused on a dozen of TFs in mouse embryonic stem cells. Ernst et al. [23] combined experimental data from a number of tissues to generate a single (global) TF-target prediction map. However, that method has also relied on PWMs and no tissue specific predictions were made. CENTIPEDE [24] used unsupervised methods to integrate TF-DNA interaction, epigenetic and evolutionary data, and is most similar to our efforts. However, CENTIPEDE relies on a footprint in the DNase data that TFs leave. Such DNase footprints are the actual locations where the TF binds and are therefore protected from DNase cleavage within the DNase HS site. Unfortunately, DNase footprint data is expensive to obtain (indeed, it was not available for most of the tissues we analyzed) since it requires very high coverage when sequencing. In addition, CENTIPEDE uses a stringent cutoff (based on PWM matching) to define putative binding sites, and thus may lose significant information in relatively weak binding sites, which have been shown to be collectively important for TF binding [71]. Neph et al. [26,72] also combined DNase footprints with PWMs to predict TF-TF interactions (though not TF-gene interactions) across a large number of human tissues, using a simple method to intersect motif matches with DHS sites. Such hard cutoffs may miss sites that score high (but just below the cutoff) for both types of data which are found by our method.

In general, we find that binding sites for the TFs we looked at are only modestly conserved when compared with controls (using PhastCons scores alone classifies ChIP-seq sequences quite poorly, see Additional file 4). This is largely consistent with the recent findings that functional non-coding sequences evolve rather rapidly [9]. As a result, adding PhastCons in the integrated model does not lead to improvements in AUC values. Nevertheless, there are a number of advantages for models that can incorporate sequence conservation. First, when DNase data is not available, adding conservation in the model leads to slight improvement over models that only use PBM data (Additional file 4). Second, the conservation of binding events can vary greatly among tissues. For example, enhancers in brain are far more constrained than those in the heart [73]. Thus it is quite possible that sequence conservation would be more informative for other ChIP-seq studies.

The application of PIPES to predict tissue specific TFBS led to results that agree well with existing knowledge regarding TF roles in specific tissues. The overall results are significantly correlated with independent gene expression data measured for these TFs across tissues even though such expression data was not used at all in our analysis.

Several extensions of our current work are possible. Recently Jiang et al. [74] reported the interesting phenomenon of sticky *k*-mers: these are *k*-mers that appear to bind to TFs with relatively high affinities in a large number of PBM experiments. The sticky *k*-mers likely represent background noises in the PBM experiments and an interesting research direction is to expand our regression method to remove such noises. In another recent study, Ballare et al. [75] reported that functional TFBSs are not always associated with high chromatin accessibility, an assumption implicitly made by us and other related methods. Rather, nucleosomes may occupy TFBSs at basal conditions, and are only remodeled or displaced upon change of cellular conditions (e.g. by hormone stimulation). Despite this unexpected relationship between TFBS and chromatin states, the paper does report that such sites, while associated with high nucleosome occupancy before stimulation, often overlap with DNase HS sites. It remains to be seen how common such cases are and what is the impact on methods such as ours that rely on DNase data to predict condition specific TF binding. Moreover, our integrative framework for utilizing additional information sources when predicting binding events on a genome wide scale could also be used for large scale comparison of different PWM methods and methods that use more complicated models to represent TF binding preferences [76-78]. This requires a detailed study and is left for future work.

## Conclusions

Combining PBM and DNase data, we presented the first major effort to provide a systematic map of computationally predicted tissue-specific targets for hundreds of TFs across a large number of tissues in mouse. We complied a resource that provides TF-target predictions for all 284 TFs studied across the 55 tissue/cell types (Supplementary Methods and Supplementary Website in Additional file 4). We believe that such comprehensive resource would be useful for both biology and computation-oriented researchers studying gene regulation [79-82].

## Methods

### K-mer based method that uses PBM data to predict TF binding

The binding specificities of TFs are often represented by position weight matrices, which assume that each position of a site contributes independently to the overall binding affinity of the site (independence assumption). The PBM data simultaneously measures binding of a TF to tens of thousands of probes, and can be used to construct a much more detailed and accurate model of TF binding specificities.

#### A biophysically-motivated model for PBM data

Our *k*-mer based PBM model (Figure 1a) is motivated by the biophysics of TF binding to the probes in PBM experiments. Following Zhao et al. [16], we denote by *Y*_*i*_ the experimentally measured intensity of the *i*-th probe on the PBM array. We denote by *F*(*i*) the (unobserved) binding probability of the TF to this probe. While these two quantities are related, due to experimental errors and scaling they are not identical. We thus assume a simple linear model for the mapping between the two:

(1) $$Y_{i}=a+\text{cF}(i)+\varepsilon_{i}$$

where *a* and *c* are constants, and *ε*_*i*_ is the error term. Since each probe is much longer than the motif itself (probe length is 36bp while motifs are generally less than 20bp with a typical length at 12bp [83]) we follow BEEML-PBM [16], and express the binding probability *F*(*i*) as the sum of the binding probabilities over all *k*-mers in the probe. Let *k* be the length of a TF binding site and *L* be the length of the variable region on the probe, we have:

(2) $$F(i)=\overset{L-k+1}{\underset{j=1}{\sum }}\lambda_{j}\cdot \beta_{S_{i}(j)}$$

where *λ*_*j*_ is the position effect at position *j* (see Supplementary Methods in Additional file 1) and $\beta_{S_{i}(j)}$ is the binding probability to *S*_*i*_(*j*), the *k*-mer at the *j*-th position of the *i*-th probe. The term *β*_*s*_ is symmetric for any *k*-mer *s*, i.e. $\beta_{s}=\beta_{\bar{s}}$, where $\bar{s}$ is the reverse complement of *s*. We note that our model allows features/k-mers to overlap, so the k-mers can be of different lengths and can contain gaps. The model relies on Lasso regression to estimate the contribution of each k-mer (see below).

By plugging in the equation of *F*(*i*) into the linear model, we can couple the different values we obtain for probe intensities as a function of the individual *k*-mer contributions, see Supplementary Methods in Additional file 1 for full details.

#### Learning the parameters of the linear model

The above linear model has approximately 4^*k*^/2 parameters (one for each *k*-mer and its reverse complement). To avoid overfitting, we use the lasso regression [84] to estimate the coefficients. Lasso is a widely used approach to linear regression that encourages a sparse model where most of the coefficients are zero. In our problem, we have the additional requirement that the coefficients must be non-negative, and this is known as positive lasso [84] (Supplementary Methods in Additional file 1). After learning the model parameters using positive lasso, we set $\beta_{s}^{\prime }=\beta_{s}/\max_{s}\beta_{s}$ to be the binding probability of the TF to the *k*-mer *s* up to a scaling constant.

Since we do not know the width of the motif bound by each TF, our method searches for *k*-mers of different lengths. In order to speed up the calculation, we first run positive lasso using all short 4–6 mers. To allow longer *k*-mers to be considered, after the first run, all pairs from the top 100 such *k*-mers (based on regression coefficients) are tested to see if the prefix of one matches the suffix of the other, yielding longer (*k* + 1)-mers. This process is repeated until up to 8-mers have been added to the feature set. In addition, we also allow for gapped *k*-mers to be considered (Supplementary methods in Additional file 1).

#### Predicting TF binding to any sequences

Our model is trained on sequences of 36bp in length (the length of the variable region of probes in PBM experiments), however, in practice, we often need to predict TF binding to longer sequences, e.g. promoter regions up to thousands of base pairs long. Searching for binding sites in long sequences often involves sliding windows with relative small size so that signals are not diluted over long regions. For simplicity, we use 36bp as our window size; otherwise, additional normalization would be needed for the PBM scores trained from 36bp probes. To predict the binding of a TF to a longer sequence (Figure 1b), we first define the binding probabilities of the TF to each overlapping 36bp region ("*site*") in that sequence:

(3) $$B=\frac{1}{B_{\text{max}}}\underset{s\in \Sigma^{k}}{\sum }\beta_{s}C_{s}$$

in which *β*_*s*_ is the binding probability to the *k*-mer *s* learned by the regression model, *C*_*s*_ is the number of times that *s* occurs in this site, and *B*_max_ is the highest possible unscaled binding probability of any 36-mer that can be achieved for the TF and is used as a scaling constant. The interpretation of this equation is that the binding probability to a 36bp sequence is the sum of binding probabilities to each of the *k*-mer of the 36bp sequence [85,86]. In practice, *B*_max_ is estimated, for each TF, from the highest unscaled binding probabilities to 100,000 randomly sampled 36bp sites. Then, the binding probability to the entire sequence is defined as the highest binding probability to any 36bp site in that sequence.

### Integrated model of TF binding *in vivo*

PBM experiments measure TF binding *in vitro*. *In vivo* binding depends on several factors including the cellular environment and the chromatin state of the bound region. In addition, it has been shown that functional TFBSs tend to be evolutionary constrained [33,34]. In this section, we describe PIPES, a method that integrates our PBM motif learning and scanning method with these additional data sources in order to determine tissue specific binding.

#### Incorporating DNase I hypersensitivity data

Let *B*_*i*_ be the probability of binding of the TF of interest to a 36bp genomic region (a *site*) indexed by *i* based on the PBM model (Equation 3), reflecting the potential of TF binding *in vitro*. We are interested in the *in vivo* occupancy of the site, denoted as *X*_*i*_. We assume that *X*_*i*_ is influenced by the chromatin state, which can be represented as a simple binary indicator variable, *A*_*i*_ (it is 1 if the chromatin is open/accessible and 0 otherwise). When the chromatin is open (*A*_*i*_ = 1), the occupancy *X*_*i*_ equals *B*_*i*_; whereas a closed chromatin at that location means that *X*_*i*_ = 0. Thus, *X*_*i*_ is simply the product of *B*_*i*_ and *P*(*A*_*i*_ = 1). The chromatin state variable can be partially determined using experimental data. Here we use DNase I hypersensitivity (HS) data (Figure 1c) which is available for several mouse and human tissues (Results). See Supplementary Methods in Additional file 1 for details.

#### The full integrated model

To further incorporate the conservation data into PIPES (Figure 1e), we consider the following graphical model:

(4) $$X_{i}\leftarrow Z_{i}\to C_{i}\to S_{i}$$

Here *X*_*i*_ is the occupancy of site *i* as described above, *Z*_*i*_ is a binary variable indicating whether site *i* is a true binding site *in vivo* or not, *C*_*i*_ is a binary variable indicating whether site *i* is conserved or not, and *S*_*i*_ is a measure of the evolutionary conservation of the site. The model assumes that true TFBSs have a higher occupancy. Similarly, when *Z*_*i*_ = 1, *C*_*i*_ is more likely to be 1 as well (a true binding site is more likely to be conserved), and this is reflected by a higher conservation score *S*_*i*_. The goal is to infer *Z*_*i*_ from the observed data *X*_*i*_ and *S*_*i*_. The evolutionary conservation measure we used is the phastCons score [35] (phastCons 46way vertebrates) downloaded from the the UCSC Genome Browser ( http://genome.ucsc.edu).

In Supplementary Methods in Additional file 1 we discuss the specific distributions we assume for each of the conditional probabilities in our model and how we learn the parameters for these distributions. After these parameters are estimated, we can compute the probability that the *i*-th site is bound by a specific TF. See Supplement for details.

### Identifying tissue-specific TF activities

We used PIPES to identify TFs likely to be active in each tissue. Intuitively, if a TF *f* is active in a tissue *T*, then the binding sites of *f* should be overrepresented in the open chromatin regions of *T*. To quantify this overrepresentation, we define *R*(*f*,*T*) as the fraction of DNase hypersensitive sites in tissue *T* that contain high-scoring binding sites of *f*. The high scoring sites are defined as those that have binding probabilities (according to the PBM model, as defined in Equation 3) higher than the top 0.1*%* of the binding probabilities for all possible sites for that TF (the exact percentage cutoff has little impact, data not shown). In practice the binding probability distribution of a TF is estimated from the 100,000 sampled sites. For each tissue, the open sites in the promoter regions are defined as those sites whose DNase tag densities are higher than 15. This threshold is chosen so that the inferred probability of chromatin being open is close to 1 according to our model. In order to identify TFs having active functions in specific cell types, we exclude the binding sites that are not tissue specific (defined as open in more than 1/3 of all tissues). Such broadly-active sites are not interesting for the purpose of finding tissue-specific TFs.

The activity score of a TF *f* in tissue *T* is defined as:

(5) $$\text{Activity}\;(f,T)=\frac{R(f,T)}{R(f,\bar{T})}$$

where $\bar{T}$ denotes all tissues other than *T*. This is used as a measure of the likely activity of the TF in that tissue. We use a simple binomial test to evaluate the significance of the activity score defined here. Suppose we observe *n* high-scoring binding sites of the TF *f* in the tissue, and among these *n* sites, *x* sites fall into DNase hypersensitive regions. By chance, the expected fraction of binding sites in the DNase HS regions is $p=R(f,\bar{T})$, thus we perform the one-sided binomial test of *x* successes in *n* trials under the null model that the probability of success is equal to *p*.

### A comprehensive collection of predicted TFBSs across 55 mouse tissues

PBM data for 284 mouse TFs were downloaded from UniPROBE [27]. DNase data for 55 mouse tissue/cell types were downloaded from the mouse ENCODE website at http://hgdownload.cse.ucsc.edu/goldenPath/mm9/encodeDCC/wgEncodeUwDnase/, and for each tissue/cell type, parameters were learned as described in Methods. A list of all the TFs and tissue/cell types is provided in Additional files 7 and 8. To predict targets of TFs, the promoter regions (+/- 10kb around transcription start sites) for all mouse genes were scanned. This choice of promoter regions is consistent with several recent publications [23,70]. We assessed the quality of our predictions in two ways. (1) The false discovery rates of top TF/tissue combinations were assessed, and in all but one of 25 combinations we examined, the FDR is below 15%. (2) For each of the 11 TFs we evaluated in the Results, we compared the set of predicted targeted genes from our method with the genome-wide targeted genes from the mouse ENCODE ChIP-seq datasets. The overlap of the two sets are highly significant for almost all TFs. See Supplementary Methods and Results in Additional file 1, Additional files 9, 10 and Supplementary Website for details.

### Comparison with other methods

To evaluate our methods we obtained ChIP-seq data for 11 TFs for which PBM data and tissue specific DNase I hypersensitivity data were available (Additional file 2). ChIP-seq data was downloaded from NCBI GEO or ENCODE using the GEO IDs or UCSC Accession IDs listed in Additional file 2.

We performed a comprehensive comparison of our PBM model with several other methods that could be or have been used in predicting TF binding on real sequences. Since most prior methods relied on PWMs, we used the PWMs reported in UniPROBE [27] for these TFs, which were obtained by applying the Seed-and-Wobble algorithm on the PBM data (S&W PWM) [11]. We also compared with BEEML [16] using both the energy matrices (BEEML Energy) and converted PWMs (BEEML PWMs), PWMs identified by RAP (RAP PWMs) [87], the max E-score of *k*-mers (Max E-score) [11], the use of occupancy score proposed by [12], a support vector regression-based method (SVR) [88], and FeatureREDUCE (unpublished). In addition, we also compared using PWMs from external sources including the JASPAR database [89] and PWMs derived from HOMER on ChIP-seq data [31]. Moreover, we also compared our integrative model with an intersection strategy that combines PWM scanning with DNase data [72] across multiple tissues, a simple method that combines PWM scanning with our DNase model, and an integrative method CENTIPEDE [24] that uses PWMs, DNase HS and conservation data. A description of the details for the settings of all methods is provided in Supplementary Methods in Additional file 1.

## Availability of supporting data

The genome-wide tissue-specific TFBS predictions for 284 mouse TFs and 55 tissue/cell types and codes for the *k*-mer based PBM modeling method are available from the supporting website at http://www.sb.cs.cmu.edu/PIPES.

## Competing interests

The authors declare that they have no competing interests.

## Authors’ contributions

SZ collected and processed the data. SZ, XH and ZBJ designed the experiments, analyzed the data and wrote the manuscript. All authors read and approved the final manuscript.

## Authors’ information

Shan Zhong and Xin He: Co-first author.

## Supplementary Material

Additional file 1 **Supplementary methods, results and figures.** This file contains Supplementary Methods, Results and Figures. Click here for file

Additional file 2 **List of TF and ChIP-seq experiments used in evaluation.** This file contains a list of information about the 11 TFs and corresponding ChIP-seq experiments used in the evaluation. Click here for file

Additional file 3 **Detailed AUCs of different methods using alternative negative sequence sets and background models (PWM-based methods).** This file lists the detailed AUCs for methods using PBM data alone, obtained when alternative negative sequence sets or background models are used. See Supplementary Methods in Additional file 1 for details. Click here for file

Additional file 4 **Details of the AUCs for comparing different methods that predict in vivo TF binding.** This file contains the AUCs of different methods that classify the positive and negative sequences from ChIP-seq experiments. Methods based on PBM data alone include S&W PWMs, BEEML PWMs, BEEML Energy, RAP PWMs, Max E-score, Occupancy score, SVR, FeatureREDUCE and our method. Methods that use external PWMs include JASPAR PWMs and HOMER PWMs. Methods based on integrative modeling that also use DNase and phastCons data include our integrative models (without and with phastCons data), baselines for our integrative models (DNase alone, phastCons alone and PBM+phastCons), simple overlapping primary S&W PWM matches with DNase, combining S&W, RAP or JASPAR PWMs with our DNase models, and CENTIPEDE under different settings. See text and Additional file 1 for details. Click here for file

Additional file 5 **Full list of the predicted tissue-specific activity score for all TFs and tissues.** This file lists the predicted activity scores and binomial test p-values for all the 284 TFs across the 55 cell/tissue types. Click here for file

Additional file 6 **List of TFs and tissues with mRNA measurement data available from **[43]**.** This file lists the TFs and tissues that have mRNA expression data for the corresponding TF available. Click here for file

Additional file 7 **List of all 55 tissues studied.** This file lists the 55 mouse tissue/cell types studied. DNase I hypersensitivity data for these tissues were downloaded from the UCSC Genome Browser. Click here for file

Additional file 8 **List of all 284 TFs in mouse with PBM data studied.** This file lists the 284 TFs in mouse studied with PBM data available. Click here for file

Additional file 9 **Overlap of genome-wide predictions with ChIP-seq data at gene level.** This file provides the number of overlapping genes in the genome-wide predictions with ChIP-seq data for the 11 TF/tissues used in the evaluations. See Supplementary Methods and Results in Additional file 1 for details. Click here for file

Additional file 10 **FDR estimates.** This file provides the estimated false discovery rates for the genome-wide predictions of the 11 TF/tissues used in the evaluation and the 15 TF/tissues with highest activity scores. See Supplementary Methods and Results in Additional file 1 for details. Click here for file
