# Supplementary material for: Predicting tissue specific transcription factor binding sites
Source: BMC Genomics. 2013 Nov 15;14:796. doi: 10.1186/1471-2164-14-796 (PMC3898213; doi:10.1186/1471-2164-14-796)
Supplement: Additional file 1 — Supplementary methods, results and figures. This file contains Supplementary Methods, Results and Figures. [file 1471-2164-14-796-S1.pdf]

Supplementary Material for Predicting tissue specific transcription factor binding sites

Shan Zhong[1,2], Xin He[1,2], and Ziv Bar-Joseph[1,3]

[1]Lane Center for Computational Biology, School of Computer Science, Carnegie Mellon University, Pittsburgh, PA 15213
[2]Co-first authors
[3]To whom correspondence should be addressed.

1 Supplementary Methods

1.1 A biophysically-motivated k-mer based model of PBM

Our k-mer based PBM model (Figure 1a in main text) is motivated by the biophysics of TF binding to the probes in PBM experiments. Following Zhao et al. [1], we denote by Y_i the experimentally measured intensity of the i-Th. probe on the PBM array. We denote by $F(i)$ the (unobserved) binding probability of the TF to this probe. While these two quantities are related, due to experiential errors and scaling they are not identical. We thus assume a simple linear model for the mapping between the two:

$$Y_i = a + cF(i) + \epsilon_i \tag{1}$$

where ϵ_i is the error term and a and c are scaling factors. Since each probe is much longer than the motif itself (probe length is 36bp while motifs are often between 6-10bp) we follow BEEML-PBM [1], and express the binding probability $F(i)$ as the sum of the binding probabilities over all possible motifs encoded by the probe. Let k be the length of a TF binding site and L be the length of the variable region on the probe, we have:

$$F(i) = \sum_{j=1}^{L-k+1} \lambda_j \cdot \beta_{S_i(j)} \tag{2}$$

where λ_j is the position effect at position j (see below for the details on the position effect) and $\beta_{S_i(j)}$ is the binding probability to $S_i(j)$, the k-mer at the j-th position of the i-th probe. The term β_s is symmetric for any k-mer s, i.e. $\beta_s = \beta_{\bar{s}}$, where $\bar{s}$ is the reverse complement of s.

Plugging in the equation of $F(i)$ into the linear model, we have:

$$Y_i = a + c\left(\sum_{j=1}^{L-k+1} \beta_{S_i(j)} \cdot \lambda_j\right) + \epsilon_i. \tag{3}$$

We can rewrite this model as:

$$Y_i = a + c\left(\sum_{s\in\Sigma^k} \beta_s X_{is}\right) + \epsilon_i \tag{4}$$

where Σ^k denotes all k-mers, and X_{is} is the number of times the k-mer s occurs in the i-th probe (weighted by the position effects):

$$X_{is} = \sum_{j:S_i(j)=s} \lambda_j \tag{5}$$

($X_{is} = 0$ if s does not occur in probe i).

Note that c and the β's are coupled in the above model and so they can not be estimated individually. Thus we can write the equation as a linear model:

$$Y_i = \beta_0 + \sum_{s\in\Sigma^k} \beta_s X_{is} + \epsilon_i \tag{6}$$

subject to the constraints that (1) $\beta_s \geq 0$ for any s; and (2) β_s is symmetric, resulting in $4^k/2$ parameters that we need to learn.

Our model differs from the PWM models of BEEML-PBM [1] since PWMs cannot capture the possibility of secondary motifs and the possible dependency of nucleotides at different positions of a motif. In our model, no such constraint is imposed, and we deal with the model complexity problem (too many parameters) through sparse linear regression (see below). However, both models use similar assumptions regarding the biophysical nature of PBM measurements and how these relate to k-mer binding probabilities. Thus our model enjoys the same benefits as those of the BEEML-PBM model: the parameters for the k-mers have clear meanings, and certain experimental artifacts (e.g. handling biases due to position and background effects) can be naturally incorporated (see below).

1.2 Position and background effects in modeling PBM data

Zhao et al. [1] considered several experimental artifacts that should be removed in order to improve the accuracy of PBM data. These include the position effect and the background effect that we adopted in our model, described briefly below. We refer to [1] for more details.

Berger et al. [2] observed that the position along a probe at which the TF binds affects the binding strength and thereby the fluorescence intensity. Zhao et al. [1] used a position effect term to explicitly model this effect. The position effect of the j-th position along a probe, denoted as λ_j, is defined as [1]

$$\lambda_j = \frac{\left\langle \frac{I_{\text{avg}}(S_{i,j})}{I_{\text{avg}}(S_i)} \right\rangle_n}{\sum_{m=1}^{L} \left\langle \frac{I_{\text{avg}}(S_{i,m})}{I_{\text{avg}}(S_i)} \right\rangle_n} \tag{7}$$

in which $I_{\text{avg}}(S_{i,j})$ denotes the average intensities of all probes containing k-mer S_i at position j, $I_{\text{avg}}(S_i)$ denotes the average intensities of all probes containing S_i at any position, and $<>_n$ denotes the average over the top n k-mers with the highest median intensities ($n = 25$).

Zhao et al. [1] observed that in a typical PBM experiment, only a small fraction of the probes have high intensities due to TF binding, and most other probes have low intensities due to background hybridization. They estimated the distribution of background intensities from the lower half of the binned distribution of all fluorescent intensities, and then the i-th probe is weighted by

$$W_i = \frac{O_i - B_i}{O_i} \tag{8}$$

in which O_i and B_i denote the observed and expected number of probes in the corresponding bin. We similarly weigh each probe by W_i in the regression model.

1.3 Positive lasso regularization and parameter learning

We implemented the positive lasso as discussed in [3] by modifying the lars package in R. To estimate the tuning parameter for the regularization terms, we used 5-fold cross validation. In order to enforce sparsity, we chose the maximum tuning parameter such that the training error of the model is within one standard deviation of the minimum training error achievable. The average running time (walltime, single thread) over the 284 TFs is 2.2 hours on a computer cluster with 2x Intel Xeon E5620 CPUs at 2.40GHz and 8GB RAM.

1.4 PBM data

The PBM data for 284 mouse TFs were downloaded from UniPROBE[4] at http://the_brain.bwh.harvard.edu/uniprobe/. For many TFs, the PBM data contains two versions of microarrays that differ in their probe designs. In such cases, for simplicity, we only run our method on the first version denoted by "v1" in the corresponding PBM dataset and report the results. In addition, for such cases we also use the trained k-mer probabilities from "v1" to predict the intensities of probes on the "v2" array.

1.5 Allowing longer and gapped k-mers

Since we do not know the width of the motif bound by each TF, our PBM model searches for k-mers of different lengths. In order to speed up the calculation, we first run positive lasso using all short 4-6 mers. To allow longer k-mers to be considered, after the first run, all pairs from the top 100 such k-mers (based on regression coefficients) are tested to see if the prefix of one matches the suffix of the other, yielding longer $(k + 1)$-mers. In addition, to allow gapped k-mers to be considered, we look at all pairs of 4- and 5-mers. If any pair of such k-mers, after connected by up to 3 gaps, appear more than once in the top $1,000$ probes with highest intensities, we add this gapped k-mer to the potential feature set as well. The entire process is repeated for three times until up to 8-mers (not counting gaps; or 10-mers with gaps) have been considered to be included in the feature set. The L1 regularization allows automatic feature selection in this process.

1.6 DNase I hypersensitivity data

The DNase I hypersensitivity data for 55 mouse tissue/cell types are downloaded from the mouse ENCODE project website at http://hgdownload.cse.ucsc.edu/goldenPath/mm9/encodeDCC/wgEncodeUwDnase/[5]. For each tissue/cell type, the "Signal" track that represents the normalized tag density is used. Chromatin in genomic regions with higher tag density are more open and therefore more accessible. For each input sequence (600bp genomic region), the tag density in the corresponding tissue at all positions are extracted, and for each overlapping 36bp window in the sequence, the max density is used as the density measurement for that window. For tissue/cell types where multiple replicates are available, the median values of all replicates are used.

1.7 Modeling DNase I hypersensitivity data and combining with PBM data

The DNase I tag densities (integer data) are modeled using a mixture of negative binomial distributions similar to [6]: each region has probability π_1 to be open ($\pi_1 = P(A_i = 1)$), and probability $1 - \pi_1$ otherwise. The tag densities of regions follow two different negative binomial distributions depending on whether $A_i = 1$ or not. To estimate the model parameters, we sample 100,000 random 36bp regions from the upstream 10kb of all genes in the data. Parameters are estimated by maximizing the likelihood function:

$$L(\mathbf{r}, \mathbf{p}, \pi_1) = P(D|\mathbf{r}, \mathbf{p}, \pi_1) = \prod_{i=1}^{N} [(1 - \pi_1) \cdot NB(D_i|r_0, p_0) + \pi_1 \cdot NB(D_i|r_1, p_1)] \tag{9}$$

in which $D = (D_1, \cdots, D_N)$ denotes the DNase I tag densities of the sampled regions, and $r_k, p_k (k = 0, 1)$ are the parameters of the negative binomial distributions of the two components. After the parameters $\phi = \{\pi_1, r_0, p_0, r_1, p_1\}$ are estimated, the probability of each site being open can be calculated by

$$P(A_i = 1|D_i) = \frac{\pi_1 \cdot NB(D_i|r_1, p_1)}{(1 - \pi_1) \cdot NB(D_i|r_0, p_0) + \pi_1 \cdot NB(D_i|r_1, p_1)}. \tag{10}$$

To combine PBM data with the DNase I data, we estimate the *in vivo* occupancy of site i as its *in vitro* occupancy estimated from the PBM data multiplied by the above probability that the site is open.

1.8 The full integrated model

To further incorporate the conservation data into the integrative model, we consider the following graphical model:

$$X_i \leftarrow Z_i \rightarrow C_i \rightarrow S_i \tag{11}$$

in which X_i is the score of the site i that combines PBM and DNase data, Z_i is a binary variable indicating whether the site i is a true binding site *in vivo* or not (hidden variable),

C_i is a binary variable indicating whether the site i is conserved or not (hidden), and S_i is a measure of the evolutionary conservation of the site. The model assumes that true TFBSs have a higher occupancy score. Similarly, when $Z_i = 1$, C_i is more likely to be 1 as well (a true binding site is more likely to be conserved), and this is reflected by a higher conservation score S_i. The goal is to infer Z_i from the observed data X_i and S_i. The evolutionary conservation measure we used is the PhastCons scores, downloaded from the the UCSC Genome Browser, that measure sequence conservation over a large number of mammalian species. For each 36bp segment, we chose the max score over the 36bp window as the PhastCons score of the sequence itself.

We model the first part ($X_i \leftarrow Z_i$) by a Beta distribution:

$$P(X_i|Z_i = k) \sim \text{Beta}(\nu_k\rho_k, \nu_k(1-\rho_k)), k = 0, 1 \tag{12}$$

where ρ_k is the mean of $X_i|Z_i = k$ and ν_k is the pseudocount for Beta distribution.

For the second part ($Z_i \rightarrow C_i \rightarrow S_i$), we define $\alpha_1 = P(C_i = 1|Z_i = 1)$ as the fraction of conserved sites among true TFBSs, and similarly $\alpha_0 = P(C_i = 1|Z_i = 0)$ as the fraction of conserved sites among non-binding sites. The conditional distribution of S_i given Z_i is:

$$\begin{aligned}
P(S_i|Z_i = 1) &= P(S_i, C_i = 1|Z_i = 1) + P(S_i, C_i = 0|Z_i = 1) && (13)\\
&= P(C_i = 1|Z_i = 1)P(S_i|C_i = 1) + P(C_i = 0|Z_i = 1)P(S_i|C_i = 0) && (14)\\
&= \alpha_1 P(S_i|C_i = 1) + (1-\alpha_1)P(S_i|C_i = 0) && (15)\\
&= \alpha_1 \frac{P(C_i = 1|S_i)P(S_i)}{P(C_i = 1)} + (1-\alpha_1)\frac{P(C_i = 0|S_i)P(S_i)}{P(C_i = 0)} && (16)\\
&= P(S_i)\left[\alpha_1 \frac{S_i}{\gamma} + (1-\alpha_1)\frac{1-S_i}{1-\gamma}\right] && (17)
\end{aligned}$$

where Equation 17 follows because according to the definition of phastCons score, $P(C_i = 1|S_i) = S_i$, and $\gamma = P(C_i = 1)$ is the total fraction of conserved sequences in the genome which can be estimated simply as the average of S_i:

$$\begin{aligned}
\gamma &= P(C_i = 1) && (18)\\
&= \sum_{S_i} P(C_i = 1|S_i)P(S_i) && (19)\\
&= \sum_{S_i} S_i P(S_i) && (20)\\
&= E(S_i) && (21)
\end{aligned}$$

Similarly,

$$P(S_i|Z_i = 0) = P(S_i)\left[\alpha_0 \frac{S_i}{\gamma} + (1-\alpha_0)\frac{1-S_i}{1-\gamma}\right] \tag{22}$$

Let $p = P(Z_i = 1)$ be the fraction of the functional TFBSs, the likelihood function over the entire dataset is given by:

$$P(X, S|\theta) \propto \prod_i \left[p \cdot \text{Beta}(X_i|\rho_1, \nu_1)P(S_i|Z_i = 1) + (1-p) \cdot \text{Beta}(X_i|\rho_0, \nu_0)P(S_i|Z_i = 0)\right]$$

$$(23)$$

where $\theta = (\rho_1, \nu_1, \rho_0, \nu_0, \alpha_1, \alpha_0, p)$ represents the model parameters. Note that the terms $P(S_i)$ are not shown because they are independent of model parameters. We estimate θ by maximizing the likelihood function for each TF and tissue/cell type on 100,000 randomly sampled sites from gene promoters. The vast majority of sequences are not bound by a TF, so we could use all the data to fit ρ_0, ν_0 and α_0 and then fix them. Thus only $(\rho_1, \nu_1, \alpha_1, p)$ are the free parameters to be estimated.

After the parameters are estimated, given any new site, its probability of being bound is predicted by

$$\frac{P(Z_i = 1|S_i, X_i, \hat{\theta})}{P(Z_i = 0|S_i, X_i, \hat{\theta})} = \frac{P(X_i|Z_i = 1, \hat{\theta})}{P(X_i|Z_i = 0, \hat{\theta})} \cdot \frac{P(S_i|Z_i = 1, \hat{\theta})}{P(S_i|Z_i = 0, \hat{\theta})}. \tag{24}$$

1.9 Comparison with other methods

To evaluate the performance of our method, we obtained ChIP-seq data for 11 TFs for which PBM data and tissue specific DNase I hypersensitivity data were available (Additional File 2). For each dataset, the top 3000 peaks with highest enrichment are extracted, and the 600bp genomic regions centered on the reported peaks are used as the positive sequences bound by the TF. Then, 600bp sequences that (1) are upstream of and (2) 300bp apart from each positive sequence, and (3) do not overlap with any other positive sequences, are used as negative sequences. For each sequence to be tested, the binding probabilities of the TF to each overlapping 36bp window (*site*) in that sequence is calculated based on the model learned (see Methods), and the maximum probability is defined as the binding probability to that sequence.

We performed a comprehensive comparison of our PBM method with several other methods that could be or have been used in predicting TF binding on real sequences, using area under the ROC curve (AUC) as the criteria. A detailed description of all methods is provided below.

1.9.1 Methods that only use PBM data

1. PWM-based methods (including S&W PWM [2], BEEML PWM [1] and RAP PWM [7]). The PWMs are converted to a log-odd scoring matrix using a zeroth-order background estimated from each input sequence respectively or a uniform background, and then all overlapping k-mers (k being the width of the PWM) on both strands are scored by the log-odd scoring matrix. The maximum score is used as the score for the sequence. In cases where a secondary PWM was reported for S&W PWMs, only the primary one is considered. For RAP PWMs, default parameters are used to extract the PWMs from PBM data.

2. BEEML Energy. The binding energy matrices reported by BEEML-PBM [1] are used to score each overlapping k-mers (k being the width of the energy matrix). The score for a k-mer is the summation of the binding energies of the corresponding bases at each

position. The minimum energy is used as the score for the sequence. Due to their same origin, BEEML Energy and BEEML PWM are considered as essentially one method.

3. Max E-score. The E-score [2] for each k-mer in the sequence is considered and the maximum E-score is used as the score for the sequence.

4. Occupancy score. The background-subtracted median intensities of all k-mers with E-scores higher than 0.35 is summed and the sum is used as the score for the sequence [8].

5. SVR. The SVR-based method [9] is run on overlapping 36bp windows in each input sequences and the maximum score is reported as the score for that sequence. The number of k-mers used is set to 1000 to speed the calculations, and default values are used for other parameters ($k = 13$ and $m = 5$).

6. FeatureREDUCE. FeatureREDUCE (unpublished yet) is among the top performing methods in a recent comprehensive evaluation of methods that infer TF binding preference from PBM data [10]. We downloaded FeatureREDUCE v1.09 from `http://bussemaker.bio.columbia.edu/software/FeatureREDUCE/`, and followed instructions provided in section 4.0 of the manual (v1.09.6 downloaded from the above URL) for training and in section 7.0 of the manual for testing, using default options and parameters.

In addition to using the 600bp sequences that are upstream of and 300bp away from the ChIP-seq peaks, we also considered two alternative sets of negative sequences including (1) randomly sampled 600bp sequences from the gene promoter regions (+/-10kbps around TSS), and (2) randomly generated 600bp sequences by RSAT [11] using the mouse genome background model.

1.9.2 Methods that use external PWMs

We included PWMs from the JASPAR database (JASPAR PWMs) [12], and PWMs derived from *in vivo* ChIP-seq data using HOMER [13] (HOMER PWMs, provided as "Custom Motif Matrices" downloaded at `http://biowhat.ucsd.edu/homer/custom.motifs`). Scoring is done in the same way as above mentioned for PWM-based methods.

1.9.3 Methods that combine sequence with DNase data

For our PIPES method, in addition to our integrated models, we also consider three baselines: (1) only using the DNase data alone, (2) only using the conservation (PhastCons) data alone, and (3) using PBM and conservation data. Moreover, to see if our DNase model can be generalized to be used with methods not based on PBM data, we also consider simple ways to combine our DNase models with PWM-based methods, by multiplying the binding probabilities predicted by the PWM models with the probabilities of the chromatin being in open status that our DNase model predicts for each k-mer, where k is the length of the PWM.

Neph et al. [14] predicted tissue-specific TF binding sites in human by overlapping motif scanning results with DNase I footprints, the actual locations bound by TFs within DNase I hypersensitive sites that are protected from DNase I cleavage [15]. In order to compare our method with this under a similar framework, we applied a similar approach by first using FIMO ([16], in the MEME suite v4.8.1) to scan the sequences for occurrences of the PWMs reported with the PBM data, and then overlapping the motif occurrences (p-value $< 1e-4$) with DNase I hypersensitivity data. When a secondary PWM is available, both PWMs are used to scan the sequences. Default parameters are used for FIMO. AUC is obtained by varying the cutoff on the DNase data.

CENTIPEDE [6] is an integrative method that can combines PWM, DNase and conservation data to predict tissue-specific TF binding. However, it is an unsupervised method and requires a user-specified cutoff on PWM match scores. To compare our method with CENTIPEDE, we used either the primary S&W PWM or the BEEML PWM of each TF to scan the upstream 10kbps of all mouse genes, assuming uniform background (the same setting as in [6]). In [6], a log odd score of $\log_2(10000)$ was used as cutoff for PWM matches. However, many BEEML PWMs are not specific and for such PWMs few sequences have scores above this cutoff. Thus we considered a variety of different cutoffs including $\log_2(100)$, $\log_2(1000)$ and $\log_2(10000)$. We also consider choosing a log odd score cutoff based on a p-value of 1e-4 of the PWM being used [17]. DNase data and phastCons scores of all positions in the PWM matches were extracted. Note that in [6] the DNase data used was strand-specific, but in our case we do not have such data available and so we did not distinguish between the two strands. Using these as training data, we learned parameters in the CENTIPEDE model for each TF. Then we scanned for PWM matches in the positive and negative sequences from the corresponding ChIP-seq data using the same cutoff, and calculated a posterior score for each PWM match using the learned parameters. For sequences with more than one PWM matches, the max posterior score is taken as the score for the sequence. Sequences with no PWM matches above the cutoff are directly classified as negative.

1.10 Overlap of genome-wide predictions with ChIP-seq data

To further evaluate our genome-wide tissue-specific binding site predictions, we examined the overlap of the target genes predicted by our method and those identified in the corresponding ChIP-seq experiments for the 11 TF/tissues we investigated. For each ChIP-seq dataset, any mouse gene that has at least one reported ChIP-seq peak within the +/-10kbps around TSS was identified as a target of the TF. For our genome-wide predictions, each gene is associated with the maximum score (PBM+DNase) within its +/-10kbps region around TSS. The top N genes from our predictions are compared with the top N genes from the ChIP-seq data (N=500, 1000, 2000 and 5000) to see the number of overlaps, and the significance of the overlaps is evaluated using permutation test with 1000 permutations.

2 Supplementary Results

2.1 Comparison of AUCs for different methods on PBM data alone

We compared our PBM method with 7 other methods that predict affinities of TF binding to given sequences based on PBM data alone. Three of these methods use PWMs or energy matrices derived from the PBM data to scan a given sequence. These include (1) the S&W PWM method which uses PWMs derived from the PBM data by the Seed-and-Wobble algorithm [2, 18], (2) the BEEML method [1], including both the BEEML Energy method which uses an energy matrix derived from the PBM data based on BEEML-PBM, and the BEEML PWM method which uses the PWM converted from the BEEML energy matrix, and (3) the RAP PWM method, which uses PWMs derived from the PBM data by RAP [7]. We also compared against methods that directly use the PBM data itself. These include the max E-score method [2] and the occupancy score method [8] which are based on a rank-based statistic called E-score derived from the PBM data. In addition, we further compared with a support vector regression (SVR) based method [9] which uses a novel string kernel to map sequences to intensities measured by PBMs, and FeatureREDUCE, one of the top performing methods in a recent large-scale benchmark study [10]. (see Supplementary Methods for details). The methods are evaluated in terms of their ability to correctly classify the positive and negative sequences for each of the 11 TFs (see Results in main text).

Results from the comparison are presented in Figures S1 and also Additional File 3. As can be seen, among methods that are based only on PBM data, for 4 of the 11 TFs including Esrra, Sox12, Pou2f1 and Crx, our PBM method achieves the highest AUC among all methods being compared. For the other TFs, the AUC by our method ranks the 3rd for Klf7 (after BEEML PWM/Energy, and Occupancy score), the 3rd for FoxA2 (after BEEML PWM/Energy, and Max E-score), the 2nd for Nkx2-5 (after Occupancy score), the 4th for Srf (after Max E-score, BEEML Energy and S&W PWM), the 4th for Ets1 (after Occupancy score, BEEML PWM/Energy, and RAP PWM), the 4th for Mafk (after PBM PWM, RAP PWM and Max E-score), and the 2nd for Max (after Occupancy score) respectively. Overall, none of the method works consistently the best across all 11 TFs being compared. Among all methods, our k-mer based PBM model has the highest average AUC for all 11 TFs of 0.731 (the 2nd is Max E-score, 0.716, p=0.1602 (one-sided paired Wilcoxon rank test); the 3rd is BEEML Energy, 0.710, p=0.1392; the 4th is RAP PWM, p=0.0337). Notably, although FeatureREDUCE worked well in the evaluation by [10], in our case it failed to run on the 600bp positive and negative sequences for 6 of the 11 TFs (Esrra, Pou2f1, Crx , Srf, Ets1 and Max), giving a NullPointerException error; and it failed to run during the training stage for Klf7, giving an error message "system computationally singular". Because of these we did not include FeatureREDUCE in Figure S1. For the other 4 TFs, its AUC is much lower than the other methods (see Additional File 3 for details). This is probably because it is not designed optimally for predicting binding to very long sequences (600bp in our case).

Two broad strategies have been developed to extract TF binding preferences from PBM data. The first strategy involves estimation of PWM using the PBM data. Berger et al. [2]

developed a rank-based statistic called E-score to quantify a TF binding strength to 8-mers, and a the constructed a PWM from the 8-mers with the highest E-scores. Zhao et al. [1] developed BEEML-PBM, a biophysical model of TF-probe binding, that directly estimates the binding energy from the PBM data while accounting for several experimental artifacts. Orenstein et al. [7] developed RAP, a method that aligns the top 8-mers whose probes have the highest intensities on the PBM array to build a PWM. The second strategy does not fit a single PWM; instead it uses information of all k-mers from the PBM data to predict TF binding to any sequences. The simplest method from this category sums over the strength (defined as median probe intensities) of all k-mers in a sequence [8]. More sophisticated methods have also been proposed. For example, Agius et al. [9] developed a support vector regression (SVR)-based method using a novel string kernel. A recent large scale comparison of dozens of methods for using PBM data has identified FeatureREDUCE and BEEML-PBM among the best methods in both reproducing *in vitro* and predicting *in vivo* binding [10]. Our result shows that none of the methods consistently work well on all TFs, and that by using our biophysically motivated model that infers binding probabilities to individual k-mers, our method is able to achieve better accuracy than the aforementioned methods on *in vivo* data in many cases.

2.2 Additional comparisons using external PWMs

We also consider using two sets of PWMs from existing databases to scan a given sequence, including (1) JASPAR PWM, which uses PWMs in the JASPAR database [12] derived from literature (Ets1 and Max), ChIP-seq data (FoxA2) or the Uniprobe database for PBM data directly (the rest TFs), and (2) HOMER PWM, which uses PWMs derived from *in vivo* ChIP-seq data using HOMER [13] (see Supplementary Methods for details). Overall, the JASPAR PWMs have similar AUCs as S&W PWMs except FoxA2 for which the JASPAR PWM was derived from ChIP-seq data. Not surprisingly, compared with methods that are based only on PBM data, scanning the sequences using HOMER PWMs (and the JASPAR PWM for FoxA2) which are derived from *in vivo* ChIP-seq data leads to much better AUC for many TFs (the average AUC for HOMER PWM is 0.770, see Additional File 3). But even so, for Esrra, Crx and Max, our PBM method outperformed HOMER PWM (Esrra: 0.943 vs 0.918; Crx: 0.783 vs 0.756; Max: 0.809 vs 0.757). Thus, *in vitro* based analysis can be a complementary resources for in-vivo studies.

2.3 Robustness of results to alternative negative sequences and background models

The above evaluation results are robust to the choice of negative sequence sets and the background model used in the PWM scoring. Using randomly sampled negative sequences from promoter or randomly generated sequences as the negative set, or using uniform background in the PWM scoring, lead to consistent results as described above. Full details are presented in the Supplementary Methods and Additional File 8.

2.4 Consistency of the model learned between PBM arrays with alternative array designs

For 98 mouse TFs, two different PBM arrays with different probe designs are available. In order to test whether the models we learned on one version (v1) of the array are stable, we used the models we learned to predict the signal intensities on the second array (v2) with a different probe design. Overall, the predicted intensities have an average Pearson correlation coefficient of 0.59 ± 0.19 with the known intensities for these TFs. Another evaluation metric we looked at is how many of our top 100 predicted probes are within the top 100 probes with highest intensities on v2. For 57 TFs, we were able to predict at least 30 of the top 100 probes within our top 100 predicted set. Since there are a total of about 44,000 probes on each array, this result is extremely significant. Therefore, our models trained on one version of the arrays are stable and work well in reproducing the intensities from the array with alternative probe design. In addition, we note that our method is primarily aimed at predicting *in vivo* data, and therefore is not optimized for reproducing the *in vitro* data. The latter aim has been extensively explored in recent literature [10].

2.5 Performance of CENTIPEDE

In order to compare the performance of our integrative model with CENTIPEDE, we trained parameters for CENTIPEDE using either the primary S&W PWM or BEEML PWM for all TFs under different cutoffs for PWM match scores, and used the learned parameters to score the 600bp positive and negative regions collected from ChIP-seq experiments (see Supplementary Methods for details). Results with a typical setting (scanning using S&W PWMs and using $\log_2(1000)$ as PWM match score cutoff) is shown in Figure 3 in the main text, and results for all other settings are shown in Additional File 3. Note that many BEEML PWMs are non-specific and hence no sequences are above certain PWM score cutoffs (the NAs in Additional File 3). See main text for more discussions.

2.6 Robustness of the tissue-specific activity scores to the percentage cutoff

When calculating the tissue-specific activity scores, a percentage cutoff is needed to define high-scoring sites by the PBM model. At different percentage cutoffs, the binding sites of a TF would obviously change and that would affect the tissue activity scores we defined. Nevertheless, the impact of this parameter is small. At the cutoff that we decided to use (top 0.1%), 5284 TF/tissue pairs have activity scores higher than 1.0, of which 2156 are higher than 1.2 and 979 are higher than 1.5. If the cutoff is increased to top 0.05%, among the above TF/tissue pairs, 4751/5284 (89.9%), 2019/2156 (93.6%) and 918/979 (93.8%) still have activity scores above 1.0, 1.2 and 1.5 respectively. We observe similar consistency when the cutoff is decreased to the top 1%. We believe that the top 0.1% cutoff that we choose is a balanced choice and the results reported are in general robust with respect to the cutoff being used.

2.7 Further evaluation of the genome-wide tissue-specific TFBS predictions

To further validate our genome-wide tissue-specific TFBS predictions, we looked at the overlap of the top target genes predicted by our method and the top genes identified by the ChIP-seq data for the 11 TF/tissues we investigated (Supplementary Methods). For the top 2000 genes that we predict, on average 24% overlap with the ChIP-seq results across the 11 TF/tissues, and all the overlaps are statistically significant (permutation p-values < 0.001). See Additional File 9 for details.

To estimate the false discovery rate for our genome-wide predictions, we scanned mouse intergenic regions that are at least 100kbps away from any known genes (excluding any k-mers containing N) for the 11 TF/tissues investigated earlier and the 15 TF/tissues in Table 1 with highest tissue activity scores [one TF/tissue (Crx/retina) is in both sets]. From this we estimated the false discovery rate in our genome wide predictions for these TF/tissues. Overall, for all except one of these 25 TF/tissues, the FDRs are below 0.15 at the score cutoff of 0.6 (the cutoff above which detailed binding site locations are reported in the prediction result files available at the Supplementary Website). Therefore, the FDR analysis supports the high accuracy of our predictions. See Additional File 10 for details.

3 Supplementary Website

Complete genome-wide prediction of binding sites for all the 284 mouse TFs with PBM data available and 55 tissue/cell types with DNase I hypersensitivity data available can be downloaded from the supplementary website at http://www.sb.cs.cmu.edu/PIPES/.

For each gene, the promoter regions (+/- 10kb around transcription start sites (TSS) that do not overlap with any other gene) are scored by the integrative model (PBM+DNase).
Column 1: TF name
Column 2: Tissue name
Column 3: Gene Ensembl ID
Column 4: Gene name
Column 5: Max score within the promoter region considered
Column 6+: When any score is higher than 0.6, all positions (relative to the TSS) with scores higher than 0.6 are listed.

In addition, BED files for predicted binding sites with scores higher than 0.6 are also provided at the Supplementary Website. These can be uploaded into UCSC Genome Browser as a custom track. Also the code for the PBM regression and a demo dataset on Crx can be downloaded from the website.

4 Supplementary Figures

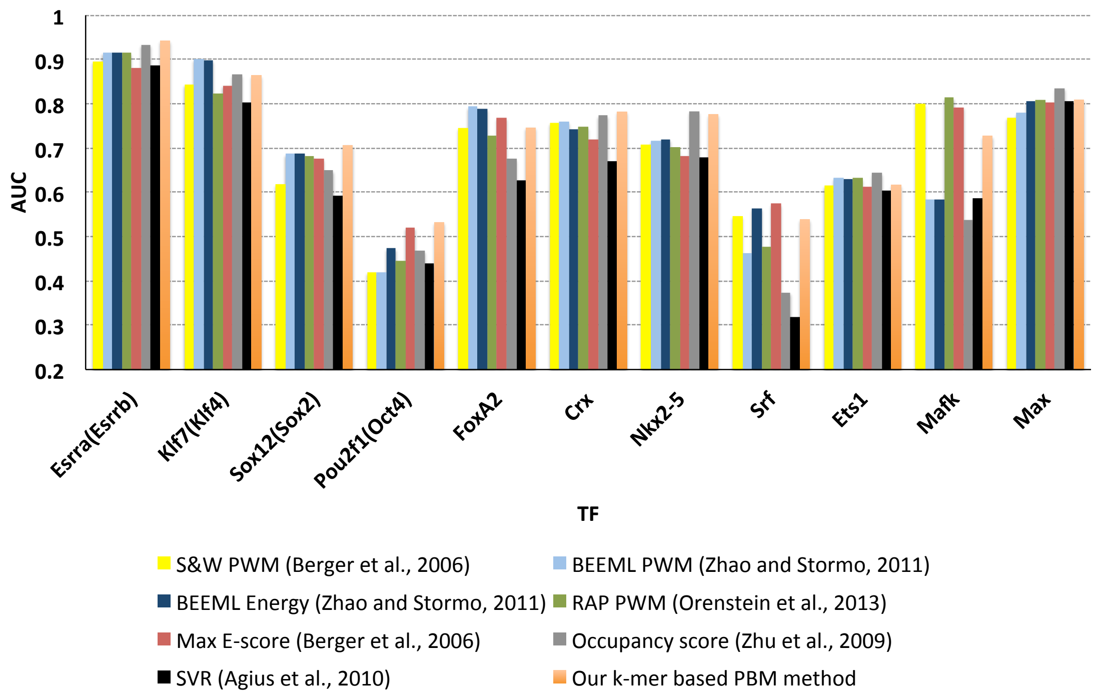

Figure S 1: Area under ROC curve (AUC) of different methods that use PBM data alone to predict *in vivo* binding sites. Shown on the x-axis are 11 TFs with ChIP-seq data available for the same TF or for a TF with a similar DNA-binding domain. In the latter case the PBM TF is shown in front and the ChIP-seq TF is shown in the parentheses. See text and Additional File 3 for details.

References

[1] Zhao Y, Stormo GD: **Quantitative analysis demonstrates most transcription factors require only simple models of specificity.** *Nat. Biotechnol.* 2011, **29**(6):480–483.

[2] Berger MF, Philippakis AA, Qureshi AM, He FS, Estep PW, Bulyk ML: **Compact, universal DNA microarrays to comprehensively determine transcription-factor binding site specificities.** *Nat. Biotechnol.* 2006, **24**(11):1429–1435.

[3] Efron B, Hastie T, Johnstone I, Tibshirani R: **Least angle regression**. *The Annals of Statistics* 2004, **32**(2):407–499.

[4] Newburger DE, Bulyk ML: **UniPROBE: an online database of protein binding microarray data on protein-DNA interactions.** *Nucleic Acids Res.* 2009, **37**(Database issue):D77–82.

[5] Mouse ENCODE Consortium: **An encyclopedia of mouse DNA elements (Mouse ENCODE).** *Genome Biology* 2012, **13**(8):418.

[6] Pique-Regi R, Degner JF, Pai AA, Gaffney DJ, Gilad Y, Pritchard JK: **Accurate inference of transcription factor binding from DNA sequence and chromatin accessibility data.** *Genome Research* 2011, **21**(3):447–455.

[7] Orenstein Y, Mick E, Shamir R: **Rap: Accurate and fast motif finding based on protein-binding microarray data**. *Journal of Computational Biology* 2013, **20**(5):375–382.

[8] Zhu C, Byers KJRP, McCord RP, Shi Z, Berger MF, Newburger DE, Saulrieta K, Smith Z, Shah MV, Radhakrishnan M, Philippakis AA, Hu Y, De Masi F, Pacek M, Rolfs A, Murthy T, Labaer J, Bulyk ML: **High-resolution DNA-binding specificity analysis of yeast transcription factors.** *Genome Research* 2009, **19**(4):556–566.

[9] Agius P, Arvey A, Chang W, Noble WS, Leslie C: **High resolution models of transcription factor-DNA affinities improve in vitro and in vivo binding predictions.** *PLoS Comp Biol* 2010, **6**(9).

[10] Weirauch MT, Cote A, Norel R, Annala M, Zhao Y, Riley TR, Saez-Rodriguez J, Cokelaer T, Vedenko A, Talukder S, DREAM5 Consortium, Agius P, Arvey A, Bucher P, Callan CG, Chang CW, Chen CY, Chen YS, Chu YW, Grau J, Grosse I, Jagannathan V, Keilwagen J, Kiełbasa SM, Kinney JB, Klein H, Kursa MB, Lähdesmäki H, Laurila K, Lei C, Leslie C, Linhart C, Murugan A, Myšičková A, Noble WS, Nykter M, Orenstein Y, Posch S, Ruan J, Rudnicki WR, Schmid CD, Shamir R, Sung WK, Vingron M, Zhang Z, Bussemaker HJ, Morris QD, Bulyk ML, Stolovitzky G, Hughes TR: **Evaluation of methods for modeling transcription factor sequence specificity.** *Nat. Biotechnol.* 2013.

[11] Thomas-Chollier M, Defrance M, Medina-Rivera A, Sand O, Herrmann C, Thieffry D, van Helden J: **RSAT 2011: regulatory sequence analysis tools**. *Nucleic acids research* 2011, **39**(suppl 2):W86–W91.

[12] Bryne JC, Valen E, Tang MHE, Marstrand T, Winther O, da Piedade I, Krogh A, Lenhard B, Sandelin A: **JASPAR, the open access database of transcription factor-binding profiles: new content and tools in the 2008 update.** *Nucleic Acids Res.* 2008, **36**(Database issue):D102–6.

[13] Heinz S, Benner C, Spann N, Bertolino E, Lin YC, Laslo P, Cheng JX, Murre C, Singh H, Glass CK: **Simple Combinations of Lineage-Determining Transcription Factors Prime cis-Regulatory Elements Required for Macrophage and B Cell Identities**. *Molecular cell* 2010, **38**(4):576–589.

[14] Neph S, Stergachis AB, Reynolds A, Sandstrom R, Borenstein E, Stamatoyannopoulos JA: **Circuitry and dynamics of human transcription factor regulatory networks.** *Cell* 2012, **150**(6):1274–1286.

[15] Galas DJ, Schmitz A: **DNAse footprinting: a simple method for the detection of protein-DNA binding specificity.** *Nucleic Acids Res.* 1978, **5**(9):3157–3170.

[16] Grant CE, Bailey TL, Noble WS: **FIMO: scanning for occurrences of a given motif.** *Bioinformatics* 2011, **27**(7):1017–1018.

[17] Touzet H, Varré JS, et al.: **Efficient and accurate P-value computation for Position Weight Matrices**. *Algorithms Mol Biol* 2007, **2**(1510.1186):1748–7188.

[18] Badis G, Berger MF, Philippakis AA, Talukder S, Gehrke AR, Jaeger SA, Chan ET, Metzler G, Vedenko A, Chen X, Kuznetsov H, Wang CF, Coburn D, Newburger DE, Morris Q, Hughes TR, Bulyk ML: **Diversity and complexity in DNA recognition by transcription factors.** *Science* 2009, **324**(5935):1720–1723.
